# Eyelid Closure at Death

**DOI:** 10.4103/0973-1075.58454

**Published:** 2009

**Authors:** Macleod AD

**Affiliations:** Department of Palliative Care, Nurse Maude Hospice, 15 Mansfield Avenue, Christchurch, New Zealand

**Keywords:** Cancer, Death, Eyelid closure, Organicity, Ptosis

## Abstract

**Aim:**

To observe the incidence of full or partial eyelid closure at death.

**Materials and Methods:**

The presence of ptosis was recorded in 100 consecutive hospice patient deaths.

**Results:**

Majority (63%) of the patients died with their eyes fully closed, however, 37% had bilateral ptosis at death, with incomplete eye closure. In this study, central nervous system tumor involvement and/or acute hepatic encephalopathy appeared to be pre-mortem risk factors of bilateral ptosis at death.

**Conclusion:**

Organicity and not psychogenicity is, therefore, the likely etiology of failure of full eyelid closure at death.

## INTRODUCTION

Surviving relatives and attending staff may associate closed eyes at death with peacefulness and restfulness, and opened eyes with discomfort or even fear. The practice of forcing eyelids closed immediately after death, sometimes using coins to lock the eyelids closed until rigor mortis intervenes, has been common in many cultures.

Open eyes at death may be interpreted as an indication that the deceased is fearful of the future, presumably because of past behaviors. Such an interpretation is invited in the newspaper headline ‘Bomber McVeigh dies, eyes wide open,’ referring to the execution of the Oklahoma City bomber.[1] A concentration camp survivor describing the awful, delirious death of a school friend during internment wrote ‘when I looked at him again he was dead… his eyes, wide open with alarm, stared into nothingness’[2] Chinese (and Taoist) belief suggests that the dead have to be judged in hell and punished according to the sins that they have committed in life.[3] In such societies the startled post-mortem appearance of fixed open eyes suggests a fearful, and not a peaceful judgement. There is virtually no modern literature on the prevalence of eye closure at death, or the causes of open eyelids post-mortem.

Shaw remarked upon this clinical sign in several of her Singaporean case vignettes.[4] Hippocrates (460-370 BC) commented upon the position of the eyelids in sleep in mortally ill persons.[5] He wrote, in respect to prognosis, that ‘the appearance of the eyes in sleep should be considered. For, if some part of the white appears when the lids are closed and it is not due to diarrhea, drug-taking nor is it the normal habit in sleep, it is a bad sign and especially fatal’. Thus in life, ptosis was considered to be a very poor prognostic sign. ‘Lid watching,’ however, is a lost medical art.[6]

The differential diagnoses of ptosis includes congenital deformity, myopathy (e.g. myasthenia gravis), oculomotor nerve palsies, age related levator dehiscence, orbitofacial trauma, eye infection, blepharospasm, conversion hysteria and cortical ‘ptosis’.[6]

A concerned and distressed Asian relative of a jaundiced patient dying of a gastrointestinal malignancy prompted the author to clinically investigate eyelid position near to and at death.

## MATERIALS AND METHODS

The eyelid positions of 100 consecutive hospice deaths attended (and death certified) by the author were recorded. In severely cachexic and dehydrated patients, as Hippocrates observed, the smaller lower eyelids may not fully close, and this was not considered to be an abnormal finding for the purposes of this study. For open eye position to be registered, the upper eyelid needs to be at least above the pupillary midline i.e. at least 50% of the white of the eye needs to be visible. Objective measures of ptosis were not performed. The presence of cranial nerve palsy, exophthalmos or a pupillary abnormality was noted.

## RESULTS

The demography of the 100 deaths is presented in Table 1. At death 63 patients had full closure of the eyelids, and 37 had eyelids open and revealing at least the lower half of the white of the eyes. No other ocular signs were evident.

**Table 1:** Demographic data of 100 consecutive hospice deaths and eyelid closure position at death

	Eyelids closed at death (%)	Eyelids open at death (%)
Number of patients	63	37
Mean age	65.4 years	63.3 years
Gender		
Male	33	18
Female	30	19
Type of cancer (primary)		
Gastrointestinal (33)	21 (64)	12 (36)
Lung (17)	10 (59)	7 (41)
Breast (12)	7 (58)	5 (42)
Hematological (8)	7 (87)	1 (13)
Melanoma (8)	4 (50)	4 (50)
Cerebral (6)	1 (17)	5 (83)
Other (7)	7 (100)	0 (0)
Non cancer	1 (50)	1 (50)

Over 80% of the small number of those who died with a cerebral tumour and 36-42% of gastrointestinal, breast and lung cancer deaths did not have eyelid closure. Clinically recognisable gross hepatic dysfunction was noted in 40% of those who died with eyes open, compared to 29% of the eye-closed group. In 30% of the eye open group, excluding those with primary cerebral tumours, there were known cerebral metastases, whereas cerebral metastases were recognised in only 16% of those who died with their eyes closed.

There appeared to be no obvious relationship between eyelid non-closure and the presence of cyanosis, medications (particularly sedatives), religious affiliation, terminal restlessness, or overt family turmoil. About 55% of persons died during “night-time’ hours (2000-0800 hours), the ‘usual’ hours of sleep. Of those who died with eyes opened, 62% died during the night. Of those who died with closed eyes, 49% died during ‘night-time’ hours, however, in severe illness the sleep-wake cycle tends to be disrupted, frequent diurnal napping is usual, and differentiating sleep and coma can be difficult.

The sign of open eyes during sleep was noted pre-mortem in many of those who died with eyes open, though consistent recording of this sign was not carried out in this clinical audit.

## DISCUSSION

The clinical audit presented has profound scientific limitations, it being a crude observational study only. The post-mortem state of opened eyes may result in significant negative consequences for the memory and reputation of the deceased. The social, cultural and personal importance of this simple clinical sign is for some tantamount, thus it is a phenomenon deserving of further study.

Direct/indirect involvement of the central nervous system, e.g. cerebral metastases or hepatic encephalopathy, appears to be a major influencing factor upon the position of the eyelids at death. Religious affiliation, terminal restlessness, medication, time of death and known (and observed) patient and family psychological and behavioral factors appear not to be clearly related.

The observation that only 63% of hospice patients died with fully closed eyes may be of surprise, but perhaps merely because of the paucity of literature. That the medical art of ‘lid watching’ is lost may account for this.[6]

Complete eye closure at death suggests peacefulness, restfulness and a comfortable ‘closure of life’. Most humans in sleep, unlike some animals such as cattle, have closed eyelids. Sleep is difficult to initiate unless bilateral eyelid ptosis, reaching the pupillary area, occurs. This occludes the visual pathway and arrests the inflow of activating sensory information.[7] Levator tone is diminished with the onset of sleep and drowsiness. An upward rotation of the eye as the lids fall further restricts the visual inflow, and enhances the prospect of sleep induction. The eyelids control the portal of entry to the principle sensory organ for perceiving the external environment, and are tightly linked to the fundamental processes of the brain itself.[7]

Complete eyelid occlusion results from the constriction of the orbicularis oculi muscles (innervated by the facial nerve) and the relocation of the levator palpebrae superior (innervated by the oculomotor nerve). The role of the lower lids in this process is less well understood. The cervical sympathetic nerves supply the Muller's muscle, but the role of this muscle in this exquisitely complex process is uncertain.

The eyelids, particularly the upper eyelids, function as a sensory gating mechanism controlled by the facial, oculomotor and sympathetic nerves, orchestrated at predominately a midbrain level, but are influenced by voluntary as well as reflex activity. Midbrain and diencephalic stimulation and lesions in man may produce eyelid opening and closure.[8] Damage to this system at several anatomical levels may result in clinical signs. Bilateral ptosis may result from damage to the levator nucleus in the dorsal portion of the oculomotor nuclear complex in the midbrain,[6] while unilateral or bilateral ptosis may occur from unilateral temporal, temporoparietal or bilateral frontal lobe disease.[9–13] Eye closure is an active process and dependent on a functional CNS. Total eye closure is usual in sleep, coma and in death.

Clemmensen has shown that in acute hepatic encephalopathy brainstem herniation is caused by increasing intracranial pressure (ICP) as a result of cytotoxic brain edema.[14] Eyewitness reports of the death of Timothy McVeigh comment upon him becoming ‘yellow’ prior to death,[1] presumably caused by the execution drug. He was undoubtedly an evil person, however, such behaviour is not the likely explanation for his dying with his eyes wide open.

Raised ICP, focal midbrain structural lesions associated with terminal cerebral tumours and hepatic encephalopathy, and not psychological or social influences, are a more compelling explanation for those who die with eyes open. Nightmares and night terrors occur during sleep (with eyes closed) indicating that eye closure does not necessarily equate to peacefulness, yet most relatives of the dead perceive this to be so. It may be that most of us die in sleep (irrespective of time of day) after having had the neurophysiological ability and energy to firstly close our eyes. Those who are unable to do so are not ‘sinners’ or ‘evil’, but rather neurologically compromised.

Further study is required to confirm that organicity and not psychogenicity is the likely etiology of failure of full eyelid closure at death.

## CONCLUSIONS

Incomplete eyelid closure pre-mortem and post-mortem is not uncommon in cancer-related deaths. It may be associated with CNS disease involvement. This startled appearance is not likely to be the consequence of ‘sins committed in life,’ but rather a neurological sign of the disease. Further research is necessary for this clinical sign at death; in some cultures it may result in significant distress for the relatives.
